# Adeno-associated virus-mediated brain delivery of 5-lipoxygenase modulates the AD-like phenotype of APP mice

**DOI:** 10.1186/1750-1326-7-1

**Published:** 2012-01-05

**Authors:** Jin Chu, Phillip F Giannopoulos, Carolina Ceballos-Diaz, Todd E Golde, Domenico Pratico

**Affiliations:** 1 Department of Pharmacology, Temple University, Philadelphia, PA, 19140, USA; 2 Center for Translational Research in Neurodegenerative Disease and Department of Neuroscience, College of Medicine, University of Florida, Gainesville, FL, USA

**Keywords:** Alzheimer's disease, animal model, amyloid beta, 5-Lipoxygenase

## Abstract

**Background:**

The 5-lipoxygenase (5LO) enzymatic pathway is widely distributed within the central nervous system. Previous works showed that this protein is up-regulated in Alzheimer's disease (AD), and that its genetic absence results in a reduction of Amyloid beta (Aβ) levels in the Tg2576 mice.

Here by employing an adeno-associated viral (AAV) vector system to over-express 5LO in the same mouse model, we examined its contribution to their cognitive impairments and brain AD-like amyloid pathology.

**Results:**

Our results showed that compared with controls, 5LO-targeted gene brain over-expression in Tg2576 mice results in significant memory deficits. On the other hand, brain tissues had a significant elevation in the levels of Aβ peptides and deposition, no change in the steady state levels of amyloid-β precursor protein (APP), BACE-1 or ADAM-10, but a significant increase in PS1, nicastrin, and Pen-2, three major components of the γ-secretase complex. Additional data indicate that the transcription factor CREB was elevated and so were the mRNA levels for PS1, nicastrin and Pen-2.

**Conclusions:**

These data demonstrate that neuronal 5LO plays a functional role in the pathogenesis of AD-like amyloidotic phenotype by modulating the γ-secretase pathway. They support the hypothesis that this enzyme is a novel therapeutic target for the treatment and prevention of AD.

## Background

The enzyme 5-lipoxygenase (5LO) catalyzes the conversion of arachidonic acid to 5-hydroxy-peroxy-eicosatetraenoic acid (5-HPETE) and subsequently to 5-hydroxy-eicosatetraenoic acid (5-HETE), which can be then metabolized into different leukotrienes [1]. The 5LO is widely expressed in the central nervous system (CNS), where it localizes mainly in neuronal cells. Its presence has been documented in various regions of the brain, including hippocampus and cortex, where significant increase in its level has been associated with aging [2-4]. Since aging is one of the strongest risk factors for developing sporadic Alzheimer's disease (AD), this pathway has been involved in its pathogenesis. To this end, 5LO immunoreactivity is increased in hippocampi of AD patients versus controls [5]; 5LO-targeted gene disruption or its pharmacologic inhibition resulted in a significant reduction of Aβ levels and deposition in the brains of the Tg2576 mice, an animal model of AD-like amyloidosis [6,7]. However, the specific mechanisms involved in this biologic effect remain unclear.

In the current study, by employing an adeno-associated viral (AAV) vector system to over-express 5LO in the brain of Tg2576, we examined its contribution to their cognitive impairments and the AD-like amyloid pathology, and investigated the mechanism responsible for it.

AAV-treated animals were tested for cognitive impairments, and brain tissues assayed for Aβ levels and APP metabolism. The results show that AAV-mediated 5LO brain gene transfer results in a worsening of their behavioral deficits and a significant increase in the amount of Aβ formed and deposited in their brains. The changes in Aβ were not associated with any significant modification of total APP, BACE-1 or ADAM-10 protein levels. By contrast, we observed that these brains had a significant increase in the levels of the transcription factor CREB, which then resulted in a significant elevation of the mRNA and protein levels for the 3 major components of the γ-secretase complex, i.e. PS1, nicastrin and Pen-2.

## Results

### Cognitive deficits in animals treated with AAV2/1 vector encoding 5LO

**Figure 1:**
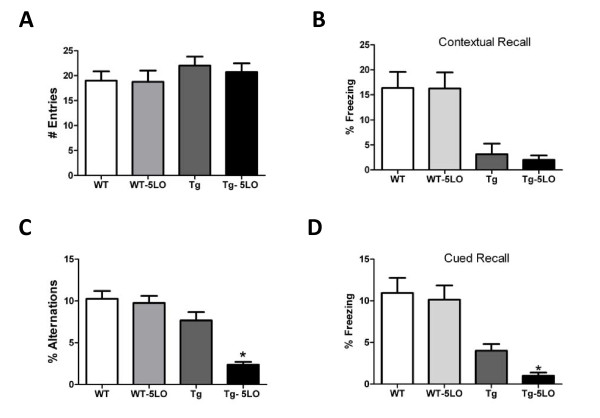
**AAV1/2-5LO over-expression modulates behavioral deficits of Tg2576 mice**. **A**. Number of total arm entries for AAV1/2-5LO and AAV-empty vector control treated Tg2576 and wild type (WT) mice. **B**. Contexual fear memory response in AAV1/2-5LO and empty vector treated Tg2576 and wild type (WT) mice. **C**. Percentage of alternations between AAV-5LO and AAV-empty vector (*p < 0.01). **D**. Cued fear memory response in AAV1/2-5LO and empty vector injected Tg2576 and wild type (WT) mice. Values represent mean ± SEM; *p < 0.01; n = 10 AAV1/2-5L0; n = 8 empty vector.

In the Y-maze, mice receiving the empty vector control and the group treated with AAV2/1-5LO did not show any significant difference in the total number of arm entries, suggesting that there were no differences in the general activity of these animals (Figure 1A). However, the number of alternations for the Tg2576 mice receiving the AAV2/1-5LO was lower when compared with their control group, and as a result the percentage of alternations was significantly reduced (Figure 1B). In the fear conditioning test, Tg2576 mice receiving the AAV2/1-5LO had generally less percentage freezing time in both the contextual and cued recall paradigms when compared with their control group. However, this difference reached statistical significance only in the cued recall (Figure 1C, D). No effect of AAV2/1-5LO on both paradigms was observed in wild type controls (Figure 1A-C).

### Aβ levels in animals treated with AAV2/1 vector encoding 5LO

**Figure 2:**
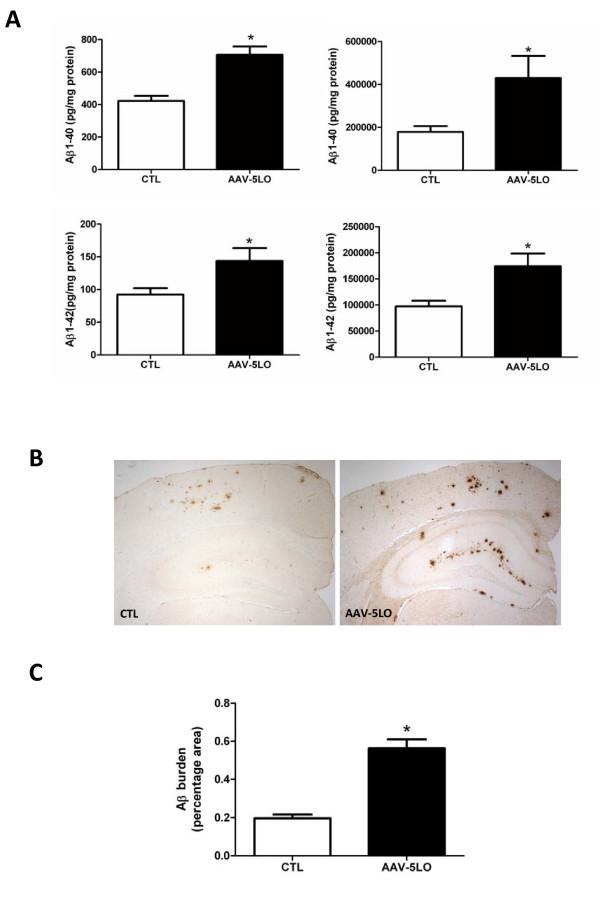
**AAV1/2-5LO over-expression modulates brain Aβ peptides levels and deposition in Tg2576 mice**. **A**. RIPA-soluble (left panels) and formic acid extractable (right panels) Aβ1-40 and Aβ1-42 levels in cortex of Tg2576 receiving empty vector (CTL) or AAV1/2-5LO gene were measured by sandwich ELISA. (n = 8 for CTL, and n = 10 for AAV-5LO; *p < 0.05). **B**. Representative sections of brains from Tg2576 mice receiving AAV1/2-5LO or empty vector (CTL) immunostained with 4G8 antibody. **C**. Quantification of the area occupied by Aβ immunoreactivity in brain of Tg2576 mice receiving AAV1/2-5LO or empty vector (CTL) (*p < 0.05). Values represent mean ± SEM (n-8 CTL; n = 10 AAV-5LO).

A week after the behavioral tests, Tg2576 mice were sacrificed and brain Aβ levels and deposition were analyzed using both biochemical and immunohistochemical methods. As shown in Figure 2A, we found that compared with Tg2576 control mice receiving the empty vector, the group treated with AAV2/1-5LO had a significant increase in the amount of RIPA-soluble and formic acid -soluble Aβ 1-40 and 1-42. These results were confirmed when a pan-Aβ antibody, 4G8, was used to detect the Aβ immunoreactive areas in the brains of these mice (Figure 2B, C).

### Over-expression of 5LO modulates neuroinflammation

**Figure 3:**
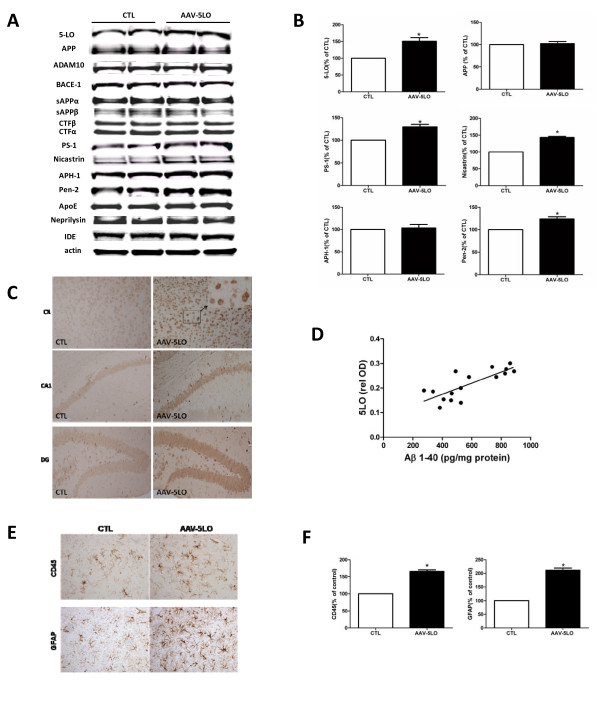
**AAV1/2-5LO over-expression alters brain APP metabolism via the γ-secretase pathway in Tg2576 mice**. **A**. Representative western blots of 5LO, APP, ADAM-10, BACE-1, sAPPα, sAPPβ, CTFα, CTF-β, PS1, Nicastrin, APH-1, Pen-2, apoE, neprilysin, IDE in brain homogenates from Tg2576 mice receiving AAV1/2-5LO or empty vector (CTL). **B**. Densitometric analyses of the immunoreactivities to the antibodies shown in the previous panel. Values represent mean ± SEM (* p < 0.01). **C**. Representative sections of different brain regions from Tg2576 mice receiving AAV1/2-5LO or empty vector (CTL) immunostained for 5-LO (× 20 magnification). **D**. Correlation between levels of 5LO protein and RIPA soluble Aβ 1-40 peptides (r^2 ^= 0.61, p = 0.0003). **E**. Representative brain sections from Tg2576 receiving AAV1/2-5LO or empty vector (CTL) immunostained for GFAP and CD45 (× 20 magnification). **F**. Quantitative analysis of the immunoreactivity for GFAP and CD45 in the same animals.(*p < 0.004).

5LO is an enzyme whose activation and expression levels have been linked to an increased inflammatory response [1]. Since neuroinflammation is also an important feature of this AD-like amyloidosis model, next we investigated the effect of 5LO over-expression on microglia and astrocytes. As shown in Figure 3D and 3E, mice over-expressing 5LO had a significant increase in the immunoreactivity for GFAP, a marker of astrogliosis, and CD45, a marker of microgliosis.

### Over-expression of 5LO modulates the γ-secretase pathway

Transgene expression was confirmed by immunoblotting and immunohistochemical approaches. Thus, we observed that 5LO protein levels were significantly higher in Tg2576 mice receiving the AAV2/1-5LO than the ones treated with the empty vector (Figure 3A, B). Immunohistochemistry analysis of the same brains showed a significant increase in neuronal 5-LO immunoreactivity when compared with mice injected with empty vector (Figure 3C). A direct correlation between Aβ levels and 5LO expression in Tg mice receiving AAV2/1-5LO was observed (Figure 3D). Since Aβ is the final product of the proteolytic processing of its own precursor, the Aβ precursor protein (APP), next we investigated whether this treatment was associated with an alteration of its expression levels. As shown in Figure 3A, B, we found that there was no difference in total APP levels between the two groups of mice. To assess the effect of AAV2/1-5LO on APP processing we investigate the steady state levels of main enzyme proteases involved: α-secretase (ADAM-10), β-secretase (BACE-1), and the four components of the γ-secretase complex by western blot analysis. As shown in Figure 3, no significant differences in the levels of ADAM-10, BACE-1, sAPPα, sAPPβ, and CTFs were observed between the two groups of mice. By contrast, we observed that mice receiving the vector encoding for 5LO had a significant increase in the steady state levels of three of the four components of the γ-secretase complex, PS1, Nicastrin, Pen-2, but not APH-1 (Figure 3A, B). In addition, we found no differences between the two groups of mice when the steady-state levels of two main Aβ degrading enzymes, neprilysisn and IDE, as well as apolipoprotein E (apoE), an Aβ chaperone, were assayed (Figure 3A).

### Over-expression of 5LO modulates transcription of the γ-secretase complex

**Figure 4:**
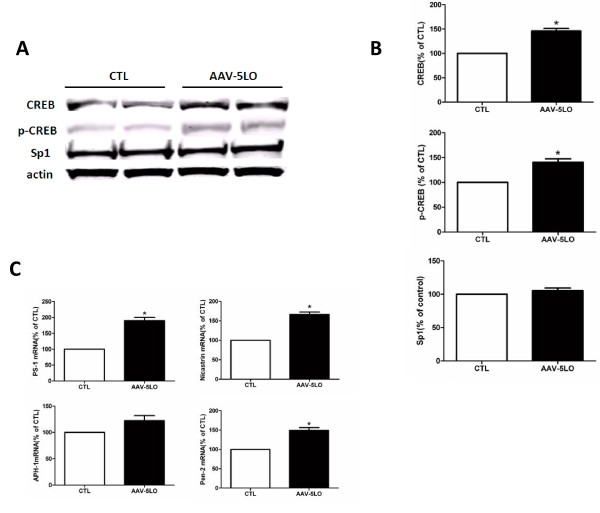
**AAV1/2-5LO over-expression modulates CREB levels and transcription of the γ-secretase complex in the brains of Tg2576 mice**. **A**. Levels of total CREB and its phosphorylated form at Ser133 and Sp1 in the cortex of Tg2576 receiving AAV1/2-5LO or empty vector (CTL) assayed by western blot analyses. **B**. Densitometric analyses of the immunoreactivities to the antibodies shown in the previous panel. **C**. Relative mRNA levels for PS1, Nicastrin, APH-1 and Pen-2 in the cortex of Tg2576 mice receiving AAV1/2-5LO or empty vector (CTL), as determined by real-time quantitative RT-PCR amplification. Values represent mean ± SEM (*p < 0.01).

The data collected so far suggest that over-expression of 5LO could modulate the γ-secretase complex expression at the transcriptional or translational level. Since previous studies have shown that pharmacological blockade of 5-LO reduces activation of the transcriptional factor CREB and then regulate the transcription of the γ-secretase complex [7], we wanted to test if this was also the case in our system. Compared with control mice, we found that Tg2576 mice treated with AAV encoding for 5LO showed a statistically significant increase in the steady state levels of total CREB and its phosphorylated form at Ser133 (Figure 4A, B). However, the same treatment did not significantly affect the steady state levels of Sp1, another transcription factor (Figure 4A, B). Additionally, quantitative real time RT-PCR analyses showed that the same mice had a significant increase in the mRNA levels for PS1, nicastrin and Pen-2 but not for APH-1 (Figure 4C).

## Discussion

The data presented in this study demonstrate that intra-cranial virally-mediated gene transfer of 5LO significantly worsens cognitive deficits, brain Aβ formation and deposition in the Tg2576 mouse model of AD and thereby provide strong new evidence that this enzymatic pathway is a modulator of amyloidogenesis in vivo.

Previous studies reported that 5LO expression is increased in rodent brain with aging, especially in the cortex and hippocampus, two regions which are known to be more susceptible to neurodegeneration. This original observation represented the basis for the hypothesis that a biologic link exists between 5LO and neurodegenerative diseases, whose strongest risk factor is aging. In line with it, 5-LO immunoreactivity is increased in hippocampi of AD patients versus controls [5], and polymorphism of the 5-LO promoter can influence the age of onset of the disease [8]. Previous work from our group have shown that genetic deficiency or pharmacologic inhibition of 5-LO results in a significant amelioration of the brain amyloidotic phenotype of the Tg2576, suggesting for the first time a functional role for this enzymatic pathway in modulating Aβ formation and deposition [6,9].

In the present study, we wanted to further extend this observation by using a virally mediated somatic gene transfer approach to increase 5LO expression levels in the central nervous system of APP mice allowing examination of its specific contribution to their cognitive deficits and brain AD-like amyloid pathology. As the somatic brain transgenic technique employed largely results in neuronal transduction, in these studies we examined the effects of 5-LO expression in neurons.

Herein, we demonstrate that its over-expression results in significantly higher Aβ peptides production and deposition in the brains of Tg2576 mice, which associated with a significant activation of both microglia and astrocytes.

In this experimental setting we observed that the increased 5LO expression had no effect on APP levels. By contrast, we observed that the APP proteolyitc processing germane to Aβ production was altered by the treatment. Thus, we found that while both ADAM-10 and BACE1 were not altered, the γ-secretase complex was significantly increased, suggesting a direct involvement of this proteolytic pathway in the observed biological effect of somatic 5-LO gene transfer. No significant changes were detected in the Aβ catabolic pathway, since no differences between the two groups were observed for neprilysin, IDE and apoE levels.

Besides these biochemical changes in the brains of the animals, we observed a worsening of their working and learning memory performances.

The first aspect was tested in the Y-maze paradigm which by recording spontaneous alternation behavior has previously been shown to be a reliable, and noninvasive test to assess some of the cognitive changes in the Tg2576 [10-12]. In the current study, we observed that over-expression of the 5LO in the brain of these mice while did not alter the number of entries, which reflects the general motor activity, it did significantly reduce the percentage of alternations during the test. These findings suggest that mice over-expressing 5LO have impairments in their immediate working memory.

In addition, animals were also tested in their learning memory ability by the fear conditioning paradigm. In accordance with the Y-maze we observed that in general AAV1/2-5LO treated mice performed worse than their controls. However, we observed a statistically significant difference only in the cued recall, but not in the contextual recall paradigm suggesting a possible amygdala involvement.

Interestingly, we did no observe any effect of AAV1/2-5LO in the wild type group, suggesting a specific effect of 5LO on the transgene.

AD is a complex neurodegenerative disease characterized by the presence of abundant intracellular and extracellular proteinaceous deposits. Aβ is a major component of these deposits and understanding of its production and clearance in the CNS is the focus of a huge effort since it can help us to develop better therapeutic approaches. The γ-secretase complex has been identified as the rate limiting enzyme for Aβ production and, for this reason, an attractive target for AD therapy.

Interestingly, in association with an increase in the steady state levels of three of its major protein components we observed that their mRNAs were also significantly elevated, suggesting a translational regulation of these genes by 5LO. Different potential transcriptional factor binding sites have been reported within their promoter regions, and among them, a CREB binding site has been shown to be essential for PS1 and Pen-2 transcriptional regulation [13,14]. In our studies we found that compared with the vehicle group, mice receiving AAV1/2-5LO had a significant increase in the levels of this transcription factor. By contrast, no differences between the two groups were observed when Sp1, another transcription factor, was assayed, suggesting a specific effect of 5LO on CREB. Taken together these findings support the hypothesis that over-expression of neuronal 5LO by modulating CREB levels results in an increased transcription of the mRNAs for three of the major components of the γ-secretase complex, which ultimately is responsible for the elevation in Aβ formation and deposition.

In summary, our studies establish a functional role for 5LO in the pathogenesis and the development of the AD-like amyloidotic phenotype, and support the hypothesis that pharmacological inhibition of this enzymatic pathway is a novel therapeutic opportunity for AD.

## Methods

### Construction of recombinant pAAV2/1 New MCS vector carrying the human 5LO gene

To obtain the target gene (5LO) from the original pcDNA2-h5LO plasmid, first we cut it with ApaI restriction endoclease, blunt end with Klenow fragment, and cut the obtained fragment with Hind3, which was isolated and purified by agarose gel electrophoresis and gel extraction kit (Qiagene). To obtain a linear vector, we cut pAAV2/1 New MCS vector with Hind3 to generate a Hind3 cohesive end, cut the fragment with EcoRV to generate a blunt end, then isolated and purified the obtained vector as above described (Qiagene). Next, we ligated 5LO DNA and the obtained linear AAV2/1 fragment with quick ligase kit (Biolab), the products of the ligation mixture were introduced into SURE2 competent cells, and transformed cells identified by genetic selection. The recombinant plasmid was prepared from these colonies and subjected to restriction endonuclease mapping to confirm the obtained DNA molecule.

### Preparation of AAV2/1 vector

The packaging, purification, and titering of the recombinant AAV2/1 vector expressing 5LO were performed as previously reported [15]. Briefly, AAV2/1 vectors expressing the 5LO under the control of the cytomegalovirus enhancer/chicken actin promoter, a woodchuck post-transcriptional regulatory element, and the bovine growth hormone, poly(A), were generated by plasmid transfection of HEK293T with helper plasmids for packing in capsid serotype 1. Forty-eight hours after transfection, cells were harvested and lysed in the presence of 0.5% sodium deoxycholate and 50 U/ml Benzonase (Sigma, St. Louis, MO) by freezing-thawing cycles, and the virus isolated by using a discontinuous iodixanol gradient, and affinity purified on a HiTrap HQ column (Amersham Biosciences). The genomic titer of each virus was determined by using quantitative real-time PCR (ABI 7900).

### Injection of AAV2/1 to neonatal mice

Tg2576 mice expressing mutant APP (KM670/671NL) gene under the control of hamster prion promoter, and their wild type littermates were previously reported [16]. Only female mice were used in this study. The injection procedures were performed as described previously [17-19]. Briefly, postnatal day 0 (P0) pups were cryoanesthetized on ice for 5 min. Two microliters of AAV2/1-5LO (1.3 × 10^13 ^genome particles/ml) were bilaterally injected into the cerebral ventricle of newborn mice using a 5 μl Hamilton syringe. The injection site was 2 mm lateral and 1 mm posterior of bregma, at a depth of 1.5 mm. The needle was left in place for 1 min after the injection was finished. A total of 5.2 × 10^13 ^viral genome (4 μl) was injected into each pup's brain. In the control group, age matched pups were injected into the cerebral ventricle with AAV2/1 empty vector. Pups were then placed on a heating pad with their original nesting material for 3-5 minutes until recovered from cryoanesthesia and then returned to their mother for further recovery. A total of eighteen pups were used for the study, ten were injected with AAV2/1-5LO and eight were injected with empty vector. Animals were then followed until they were 13-month-old, when they first underwent to behavioral testing. During this time mice did not display any significant difference in their general health, growth and weight gain whether they were randomized to the empty vector or the AAV2/15-LO treatment groups. Two weeks after the behavioral testing, mice were sacrificed. Macroscopic analysis of their major organs (liver, intestine, spleen, heart, brain) did not reveal any significant difference between the two groups. All animal procedures were approved by the Institutional Animal Care and Usage Committee, in accordance with the U.S. National Institute of Health guidelines.

### Behavioral tests

All animals were pre-handled for 3 days prior to testing. They were tested in a randomized order, and all tests conducted by an experimenter blinded to the treatment.

### Fear-conditioning

Two weeks before sacrifice, fear conditioning experiments were performed following methods previously described [20,21]. Briefly, tests were conducted in a conditioning chamber (19 × 25 × 19 cm) equipped with black methacrylate walls, transparent front door, a speaker and grid floor (Start Fear System; Harvard Apparatus). On day one, mice were placed into the conditioning chamber and allowed free exploration for 2 min in the white noise (65 Db) before the delivery of the conditioned stimulus (CS) tone (30 s, 90 Db, 2000 Hz) paired with a foot-shock unconditioned stimulus (US; 2 s, 0.6 mA) through a grid floor at the end of the tone. A total of 3 pairs of CS-US pairing with a 30 s inter-trial interval (ITI) were presented to each animal in the training stage. One min after the last foot-shock, the mouse was removed from the chamber and placed back in its home cage. The contextual fear-conditioning stage started 24 hrs after the training phase when the animal was put back inside the conditioning chamber for 5 min with white noise only (65 dB). During this time the animal's freezing responses to the environmental context were recorded. The tone fear-conditioning stage started 2 hrs after the contextual phase. The animal was placed back to the same chamber with different contextual cues, including white wall, smooth metal floor, lemon extract drops, and dimmed yellow light conditions. After 3 min of free exploration, the mouse was exposed to the exactly same 3 CS tones with 30 s ITI as in the training session, without the foot-shock and its freezing responses to the tones were recorded. One minute after the last tone, the mouse was brought back to the home cage.

### Y-maze

The Y-maze apparatus consisted of three arms 32 cm (long) ×10 cm (wide) with 26-cm walls (San Diego Instruments). Testing was always performed in the same room and at the same time to ensure environmental consistency. Briefly, each mouse was placed in the center of the Y-maze and allowed to explore freely through the maze during a 5-min session. The sequence and total number of arms entered were video recorded. An entry into an arm was considered valid if all four paws entered the arm. An alternation was defined as three consecutive entries in three different arms (i.e. 1,2,3 or 2,3,1, etc). The percentage alternation score was calculated using the following formula: Total alternation number/total number of entries-2)*100. Furthermore, total number of arm entries was used as a measure of general activity in the animals. The maze was wiped clean with 70% ethanol between each animal to minimize odor cues.

### Immunoblot analyses

Proteins were extracted in EIA buffer containing 250 mM Tris base, 750 mM NaCl, 5% NP-40, 25 mM EDTA, 2.5% Sodium Deoxycholate, 0.5% SDS and an EDTA-free protease inhibitor cocktail tablet (Roche Applied Science), sonicated, centrifuged at 13,000 rpm for 45 min at 4°C, and supernatants used for immunoblot analysis, as previously described [6,20]. Total protein concentration was determined by using BCA Protein Assay Kit (Pierce, Rockford, IL). Samples were electrophoretically separated using 10% Bis-Tris gels or 3-8% Tris-acetate gel (Bio-Rad, Richmond, CA), according to the molecular weight of the target molecule, and then transferred onto nitrocellulose membranes (Bio-Rad). They were blocked with Odyssey blocking buffer for 1 hr; and then incubated with primary antibodies overnight at 4°C. After three washing cycles with T-TBS, membranes were incubated with IRDye 800CW or IRDye 680CW-labeled secondary antibodies (LI-COR Bioscience) at 22°C for 1 hr. Signals were developed with Odyssey Infrared Imaging Systems (LI-COR Bioscience). Actin was always used as an internal loading control. Primary antibodies used were as follows: anti-APP N-terminal raised against amino acids 66-81 for total APP (22C11; Chemicon Int.), anti-BACE-1 (IBL America), anti-ADAM-10 (Chemicon Int.), anti-PS1 (Cell Signaling), anti-nicastrin (Cell Signaling), anti-Pen2 (Invitrogen), anti-APH-1 (Millipore); anti-sAPPα (2B3; IBL America); anti-sAPPβ (6A1, IBL America); anti-CTFs (EMD Biosciences Inc.); anti-neprilysin (Santa Curz); anti-IDE N-terminal (EMD Biosciences Inc.); anti-apoE (Santa Cruz); anti-CREB and anti-p-CREB (Cell Signaling); anti-Sp1 (Cell Signaling); anti-5-LO (BD Bioscience), anti-β actin (Santa Cruz). IRDye infrared secondary antibodies were from LI-COR Bioscience.

### Biochemical analyses

Mouse brain homogenates were sequentially extracted first in RIPA for the Aβ 1-40 and 1-42 soluble fractions, then in formic acid for the Aβ 1-40 and 1-42 insoluble fractions, and then assayed by a sensitive sandwich ELISA kits (WAKO Chem.) as previously described [22,23].

### Quantitative real time RT-PCR

RNA from cortex tissue of mice was extracted and purified using the RNeasy mini-kit (Qiagen), and used as previously described [24]. Briefly, 1 μg of total RNA was used to synthesize cDNA in a 20 μl reaction using the RT^2 ^First Strand Kit for reverse transcriptase-PCR (Super Array Bioscience). Mouse PS1, nicastrin, APH-1, Pen-2 genes were amplified by using the corresponding primers obtained from Super Array Bioscience. β-actin was used as an internal control gene to normalize for the amount of RNA. Quantitative real-time RT-PCR was performed by using Eppendorf^® ^ep realplex thermal cyclers (Eppendorf, NY). Two μl of cDNA was added to 25 μl of SYBR Green PCR Master Mix (Applied Biosystems, CA). Each sample was run in duplicate, and analysis of relative gene expression was done by using the 2^-ΔΔCt ^method [25]. Briefly, the relative change in gene expression was calculated by subtracting the threshold cycle (ΔCt) of the target genes (PS1, nicastrin, APH-1 and Pen-2) from the internal control gene (β-Actin). Based on the fact that the amount of cDNA doubles in each PCR cycle (assuming a PCR efficiency of 100%), the final fold-change in gene expression was calculated by using the following formula: relative change = 2^-ΔΔCt^.

### Immunohistochemistry

Immunostaining was performed as reported previously by our group [20,23]. Serial 6-μm-thick coronal sections were mounted on 3-aminopropyl triethoxysilane (APES)-coated slides. Every eighth section from the habenular to the posterior commissure (8-10 sections per animal) was examined using unbiased stereological principles. The sections for Aβ assay were deparaffinized, hydrated, pretreated with formic acid (88%) and subsequently with 3% H_2_O_2 _in methanol. The sections for 5-LO assay were deparaffinized, hydrated and treated with 3% H_2_O_2 _in methanol, and subsequently retrieved antigen with citrate (10 mM). Sections were blocked in 2% fetal bovine serum before incubation with primary antibodies (4G8 for Aβ, and monoclonal anti-5LO antibody for 5LO, anti-GFAP, anti-CD45) overnight at 4°C. Subsequently, sections were incubated with the appropriate biotinylated anti-mouse IgG (Vector Lab.) and then developed by using the avidin-biotin complex method (Vector Lab.) with 3,3'-diaminobenzidine (DAB) as a chromogen. Light microscopic images were used to calculate the area occupied by Aβ-immunoreactivity using the software Image-Pro Plus for Windows version 5.0 (Media Cybernetics). The threshold optical density that discriminated staining from background was determined and kept constant for all quantifications. The area occupied by Aβ-immunoreactivity was measured by the software and divided by the total area of interest to obtain the percentage area of Aβ-immunoreactivity.

### Data analysis

One-way ANOVA followed by the Bonferroni's Multiple Comparison tests was performed using GraphPad Prism 5.0. All data are presente'd as mean ± S.E.M. Significance was set at p < 0.05.

## List of abbreviations

AD: Alzheimer's disease; 5-LO: 5-lipoxygenase; Aβ: amyloid beta; AAV: adeno-associated virus; APP: amyloid beta precursor protein; BACE-1: beta secretase-1; PS1: presenilin 1; sAPPα: secreted APPalpha; sAPPβ: secreted APP beta.

## Competing interests

The authors declare that they have no competing interests.

## Authors' contributions

JC, PG, CC-D performed the experiments. JC, TEG, DP analyzed the results. DP designed the study and wrote the manuscript. DP is the principal investigator. All authors read and approved the final manuscript.
